# Reviewing the availability, efficacy and clinical utility of Telepsychology in dialectical behavior therapy (Tele-DBT)

**DOI:** 10.1186/s40479-021-00165-7

**Published:** 2021-10-30

**Authors:** Hanneke van Leeuwen, Roland Sinnaeve, Ursula Witteveen, Tom Van Daele, Lindsey Ossewaarde, Jos I. M. Egger, Louisa M. C. van den Bosch

**Affiliations:** 1 Vincent van Gogh Centre of Excellence for Neuropsychiatry, Vincent van Gogh Institute for Psychiatry, Stationsweg 46, 5803 AC Venray, the Netherlands; 2 Cognition and Behaviour, Donders Institute for Brain, Radboud University, Nijmegen, the Netherlands; 3 Dialexis, Training institute for Dialectical Behavior Therapy, Nijmegen, The Netherlands; 4 UPC KU Leuven, Kortenberg, Belgium; 5 Department of Neurosciences, Mind Body Research, KU Leuven, Leuven, Belgium; 6 GGNet for Psychiatry, Apeldoorn, the Netherlands; 7 Dutch Centre for treatment in DBT (NB-DBT), Harderwijk, the Netherlands; 8 Expertise Unit Psychology, Technology & Society, Thomas More University of Applied Sciences, Antwerp, Belgium; 9 Centre for Anxiety and Obsessive-Compulsive Disorders, Vincent van Gogh Institute for Psychiatry, Venray, the Netherlands; 10 Stevig Specialized and Forensic Care for People with Intellectual Disabilities, Dichterbij, Oostrum, The Netherlands

**Keywords:** Telehealth, Telemedicine, COVID-19, Phone consultation, Dialectical behavior therapy

## Abstract

**Background:**

Telepsychology is increasingly being implemented in mental health care. We conducted a scoping review on the best available research evidence regarding availability, efficacy and clinical utility of telepsychology in DBT. The review was performed using PRISMA-ScR guidelines. Our aim was to help DBT-therapists make empirically supported decisions about the use of telepsychology during and after the current pandemic and to anticipate the changing digital needs of patients and clinicians.

**Methods:**

A search was conducted in PubMed, Embase, PsycARTICLES and Web of Science. Search terms for telepsychology were included and combined with search terms that relate to DBT.

**Results:**

Our search and selection procedures resulted in 41 articles containing information on phone consultation, smartphone applications, internet delivered skills training, videoconferencing, virtual reality and computer- or video-assisted interventions in DBT.

**Conclusions:**

The majority of research about telepsychology in DBT has focused on the treatment mode of between-session contact. However, more trials using sophisticated empirical methodologies are needed. Quantitative data on the efficacy and utility of online and blended alternatives to standard (i.e. face-to-face) individual therapy, skills training and therapist consultation team were scarce. The studies that we found were designed to evaluate feasibility and usability. A permanent shift to videoconferencing or online training is therefore not warranted as long as face-to-face is an option. In all, there is an urgent need to compare standard DBT to online or blended DBT. Smartphone apps and virtual reality (VR) are experienced as an acceptable facilitator in access and implantation of DBT skills. In addition, we have to move forward on telepsychology applications by consulting our patients, younger peers and experts in adjacent fields if we want DBT to remain effective and relevant in the digital age.

**Supplementary Information:**

The online version contains supplementary material available at 10.1186/s40479-021-00165-7.

## Background

Telepsychology,^1^ i.e. the provision of psychological services using telecommunication or digital technologies (e.g. internet, telephone applications and virtual reality), is on the rise [2–6]. COVID-19 accelerated this trend [7]. However, a scoping review about the efficacy and clinical utility of telepsychology in DBT is lacking. The aim of the current review is to help DBT-therapists make empirically supported decisions about the use of telepsychology during and after the current pandemic and to anticipate the changing digital needs of patients and clinicians.

Standard DBT is an empirically supported, cognitive behavioral treatment for adults and adolescents suffering from chronic suicidal and self-harming behavior, characteristic for borderline personality disorder (BPD) [8, 9]. DBT is a comprehensive program that consists of four primary modes of treatment delivery, to address the multiple needs of suicidal patients [9–11]. In individual therapy, patients figure out how they can take realistic steps to a life worth living and how to stay motivated. In skills training, the focus is on acquiring skills from trainers and peers. Consultations outside of office hours, preferably by the individual therapist, facilitate generalization of skills to situations where patients need them the most, e.g. during suicidal crises. Even though this mode is often called “phone consultation”, DBT-teams have used all kinds of technology to stay in touch with clients [12]. The therapist consultation team, lastly, helps individual therapists and skills trainers to deliver adherence treatment and remain motivated throughout the process [9, 11].

Although primarily designed for suicidal and self-harming behavior, DBT remains effective when tailored to fit the needs of other clinical populations, age groups, or treatment settings [13, 14]. A significant part of this customizability relates to the underlying treatment rationale, Marsha Linehan’s biosocial theory. This theory explains maladaptive behavior, including suicidal behavior and non-suicidal self-injury (NSSI), as manifestations of pervasive emotion dysregulation or as ways of coping with it [9, 15]. Emotion regulation capacity is a transdiagnostic and dimensional construct, assumed to play a key role in a broad range of mental illnesses [15–18]. Especially the DBT skills training is evolving from a treatment mode for suicidal patients with BPD to a transdiagnostic intervention [9]. Telepsychology is increasingly being used in DBT supervision and -training for professionals [19–25] and in mental health care in general [26, 27]. Arguments both in favor and against such an evolution can be made. On the one hand, evidence suggests that the efficacy of telepsychology is comparable to face-to-face care in diverse populations and settings [28–30]. On the other hand, studies about telepsychology have primarily focused on short-term effects [31–33]. In addition, there is insufficient research to consider any telepsychology intervention as evidence-based for suicidal ideation, self-harm or BPD [34, 35]. However, a comprehensive overview of research regarding the use of telepsychology in DBT is lacking.

We conducted a scoping review to fill this research gap [36]. Since we are interested in the use of telepsychology in DBT in all of its capacities and welcome the evolution toward dimensional theoretical constructs in psychiatry, we did not restrict ourselves to one specific diagnostic category. The overarching aim was to document the best available research evidence regarding the efficacy and clinical utility of telepsychology in DBT. In doing so, we used the definitions of the American Psychological Association (APA) [37, 38]. *Treatment efficacy* referred to the scientific evaluation of whether a treatment works. *Clinical utility* comprised the applicability, feasibility, and usefulness of the intervention in specific situations, as well as the generalizability of an intervention whose efficacy had been established [37, 38]. To obtain a detailed overview, we reviewed all types of research evidence (i.e. clinical opinion, observation, consensus among recognized experts, systematized clinical observation, quasi experiments, randomized controlled trials) that could help answer our research questions. At the same time, we took the difference in methodological quality into account when interpreting the results [39, 40]. Within our overarching aim, we had three specific research questions (RQ):

RQ1:What do we know about the efficacy and clinical utility of telepsychology in standard DBT, i.e. using telecom for between-session-contact, in support of skills generalization?

RQ2: To what extent is telepsychology equivalent or superior to face-to-face contact in other modes of DBT treatment, i.e. individual therapy, skills training, consultation team?

RQ3: Does the addition of telepsychology to standard DBT modes, −strategies, −procedures and skills increase the efficacy or clinical utility of DBT?

## Methods

### Criteria and identification of studies

**Fig. 1:**
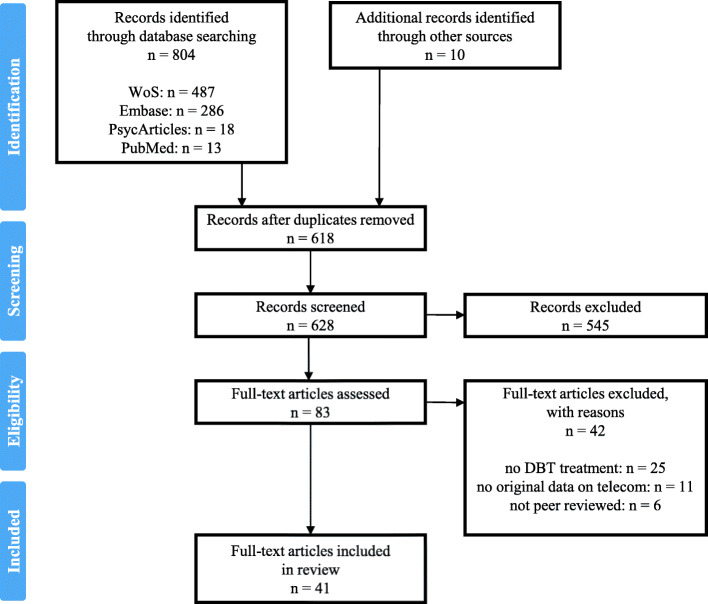
PRISMA Flow Diagram of literature search and selection

A search was conducted in PubMed, Embase, PsycARTICLES and Web of Science (WoS) until the 9th of March 2021, following PRISMA-ScR guidelines [36]. Search terms for telepsychology were included and combined with search terms that relate to DBT. Search terms (Additional file 1) and syntax (Additional file 2) were modified as necessary for each database. Only 1) English, French, German and Dutch manuscripts from 2) peer-reviewed sources, that 3) provided quantitative or qualitative research evidence 4) on efficacy or clinical utility of 5) the use of digital technology 6) in DBT treatment or 7) implementation of telepsychology in DBT treatment were considered. To identify other potentially relevant studies we 1) crosschecked the reference lists and citing articles of identified studies, 2) checked the references of *The Oxford Handbook DBT* [13], and 3) screened the references of *Phone Coaching in Dialectical Behavior Therapy* [12] (Fig. 1).

### Selection of studies

All records from the database searches were imported in EndNote. This collection was electronically and manually deduplicated. The titles and abstracts were screened for eligibility by two review authors independently (HvL, RS). Records that clearly did not fulfill the criteria were excluded. The remaining references were made available in full text and assessed for eligibility by the same two review authors (HvL, RS) (Additional file 3). Conflicting decisions were discussed until agreement was reached. If necessary, we consulted with two other authors (UW, LvdB).

For the randomized controlled trials (RCTs) and pre-post studies, detailed descriptions of design, participants, experimental and comparator interventions, measures, and statistical significance of changes in the primary outcome variables can be found in Table 1. If an effect was significant, we also reported Cohen’s d or calculated it ourselves, following the formulas and procedures mentioned in Lakens et al. [41]. In the results section, we go over the main findings per research question and per technology.

## Results

### Sample of studies

The original database search yielded 804 reports. Cross-checking and screening *The Oxford Handbook DBT* and *Phone Coaching in Dialectical Behavior Therapy* resulted in 10 records. This sample of 814 records contained 196 duplicates. After screening titles and abstracts, 83 references were selected as potentially eligible, of which 41 clearly met the inclusion criteria (Fig. 1). A detailed overview of the 83 full-text articles that we assessed, and the reason why specific articles were excluded, can be found in Additional file 3.

### Study characteristics

In total, 41 studies were selected. The majority of the studies consist of observational data, secondary analyses, focus groups and surveys, patients’ opinions and expert opinions. Eighteen out of forty-one studies focused on the frequency, efficacy and utility of phone consultation. Nine studies focused on the added value of smartphone applications. Nine studies focused on internet delivered skills training. Four studies focused on virtual reality in DBT. One study focused on a computer program and one study focused on learning a DBT skill by means of video. All studies were in the initial stage of clinical research (i.e. feasibility, acceptance, usability).

**Table 1:** Summary of the primary outcomes of randomised controlled trials (RCTs) and pre post studies using classical statistics

Study	Design	Participants	Technology	Experimental intervention	Comparator Interventions	Measures	Cohen’s d	Test-scores & *p*-values
*Lopez (2020)* [42, 43]	Post-only	Adults diagnosed with depression, bipolar disorder or anxiety disorder. N: 15 + 20 Age *M (SD):* 40 (16)	Videoconferencing	Two **weekly DBT skills training group online** via Video Teleconferencing (VTC)	Two weekly DBT skills training group face to face	*Efficacy* RCCS *Utility* Satisfaction		*t* (24) = 3.7, *p* = .001 *t* (24) = 1.39, *p* = .19
*Lungu* (2015) [44]	Pre-post	Adults with a wide range of psychopathology N: 25 Age M (SD): 44 (15)	Internet delivered skills training	An 8 week **online trans-diagnostic DBT skills training** for Emotion Regulation (iDBT-ER)	None	Efficacy DBT-WCCL DERS AAQ PHQ-9 OASIS KIMS OQ-45 Utility CSQ	*d* = 1.11 n.c. n.c *d* = 0.69 *d* = 0.89 *d* = 0.02 *d* = 1.00	*F*(1, 45.32) = 28.67, *p* <. 01 *F*(1, 42.73) = 30.53, *p* < .01 *F*(1, 23.64) = 21.98, *p* < .01 *F*(1, 24.35) = 22.20, *p* < .01 *F*(1, 44.30) = 9.05, *p* < .01 *F*(1, 22.49) = 18.38, *p* <. 01 *F*(1, 24.05) = 22.55, *p* <. 01
*Navarro-Haro (2019)* [45]	Pre-post	Adults diagnosed with a Generalized Anxiety Disorder according to the MINI N: 19 Age M (SD): 44 (10)	Virtual Reality	Six one hour Mindfulness Based Intervention (MBI) group sessions plus six ten minutes additional **Virtual Reality** Mindfulness Skills Training (VR DBT) individual	None	*Efficacy* GAD-7*	*d* = 1.33	*β* = − 4.38, *p* < .001
*Rizvi* (2011) [46]	Pre-post	Adults diagnosed with BPD and had a comorbidity with substance abuse disorders who had been in DBT treatment for at least 2 months, and their therapists. N: 22 Age M (SD): 34 (10)	Mobile (web) Application	10 to 14 days access to **DBT coach application** for smartphones, to enhance generalization of the emotion regulation skill of opposite action in an interactive way, as an add-on for patients who were participating in a standard DBT program	None	Efficacy DBT Coach Data^a^ (a) emotional intensity (b) urges to use substances Daily assessments for patients^b^ (a) urge to use substances (b) how helpful is the DBT Coach app (c) how helpful is the Opposite Action skill Confidence in executing opposite action^c^ BDI BSI “Therapist Questionnaire”^d^ (a) patients skill use in general (b) the use of the opposite action skill before and after using the DBT Coach application (c) number of phone calls outside the sessions	a) *d* = − 1.32 b) *d* = − 0.90 a) *d* = − 0.48 *d* = 0.59 *d* = 0.55 *d* = 0.43 b) *d* = − 0.84	a) *t* (21) = − 6.17, *p* < .001 b) *t* (21) = − 4.22, *p* < .001 a) *t* (21) = − 2.26, *p* = .035 b) *t* (21) = − 1.14, *p* = .269 c) *t* (21) = .67, *p* = .508 *t* (21) = 2.93, *p* = .008 *t* (21) = 2.69, *p* = .014 *t* (21) = 2.49, *p* = .021 a) *t* (21) = − 1.06, *p* = .30 b) *t* (21) = − 2.79, *p* = .01 c) *t* (21) = 1.15, *p* = .26
*Rizvi* (2016) [47]	Pre-post	Adults diagnosed with BPD and had a recent history of repeated nonsuicidal self-injury (NSSI) N: 16 Age *M* (*SD*): 28 (8)	Mobile (web) Application	**DBT coach application** for smartphones, a mobile web app to enhance generalization of mindfulness, emotion regulation, interpersonal effectiveness and distress tolerance skills in an interactive way, as an add-on for patients who were participating in a standard DBT program	None	*Efficacy* GAF GSI BSI DERS SITB DBT-WCCL		n.s. “ “ “ “ “ “
*Salamin (2020)*	Pre-post	Adults diagnosed with BPD who 1) were participating in a standard DBT program in the period of interest (i.e. 8 weeks before confinement and 8 weeks during confinement) and 2) filled out the online diary cards. N: 7 Age *M (SD)*: 35 (12)	Telephone consultation and videoconferencing	6 weeks of **individual therapy and DBT skills training by means of telephone consultation or videoconferencing.** Skills training was limited to 45 min (instead of two hours) per week and was provided individually. Adaptions were necessary to continue standard DBT during lockdown.	None	*Efficacy* #Suic. behaviour/ week #NSSI/ week #Alcohol/ week #Binge-eating/ week Suic. ideation/ day Fear/ day Shame/ day Tension/ day Distress/ day	n.c. n.c. *d* = − 0.42 *d* = − 0.34 *d* = − 0.01 *d* = − 0.17	n.s. n.s. F(1, 63.1) = 3.1, *p* = .08 F(1, 63.5) = 4.3, *p* = .04 β = − 0.00, *p* = .964 β = − 0.59, p < .001 β = − 0.48, p < .001 β = − 0.35, *p* = .029 β = − 4.57, *p* = .030
*Schroeder* (2018) [48]	Pre-post	Adults diagnosed with depression (52%), General Anxiety Disorder (48%), Borderline Personality Disorder (40%), PTSD (27%), Bipolar Disorder (14%) N Tot: 73 Age *M*: 37	Mobile (web) Application	**28 days of semi-personalized text messages** in the morning to motivate people to use **“Pocket Skills”,** a mobile web app containing written information, videos, and a conversational agent (avatar of M. Linehan) offering information on the basics of DBT, DBT skills and a DBT diary card.	None	*Efficacy* OASIS PHQ-9 DBT-WCCL	n.c. n.c. n.c.	*β* = − 0.66, *p* < .001 *β* = − 0.79, *p* < .001 *β* = − 0.06, *p* < .001
*Waltz (2009)* [49]	RCT Cross-over	Adults diagnosed with BPD according to SCID II, naïve to DBT N: 15 + 15 Age *M* (*SD*): 33 (9)	Video	**Video** of Marsha Linehan teaching the ‘opposite action’ skill step by step from the emotion regulation module of DBT.	Episode from a serie called *The Desert Speaks* on the culinary, medicinal and scientific uses of pepper plants.	*Efficacy* OAKQ	*d* = 0.8	*F* = 18.74, *p* < .001
*Wilks (2018)* [50] Wilks (2017) [51] Wilks (2020)	RCT Cross-over	Adults diagnosed with BPD that show suicidal behaviour and heavy episodic drinking. N: 29 + 30 Age *M* (*SD*): 38 (10)	Internet delivered skills training	**Internet-delivered DBT skills training** (8 sessions, consisting of mindfulness, reducing problematic drinking, emotion regulation and distress tolerance) and daily emails and/or text messaging for motivation.	Waiting list	*Efficacy* SSI AUDIT TLFB DERS	n.c.	*β* = − 1.08, *p* = 0.22 *β* = − 2.05, *p* = 0.09 *β* = 0.81, *p* = 0.02 *β* = − 4.57, *p* = 0.18

*RCCS* Rovai Community Connection Scale, *DBT-WCCL* Dialectical Behavior Therapy Ways of Coping Checklist, *DERS* Difficulties in Emotion Regulation Scale, *AAQ* Acceptance and Action Questionnaire, *PHQ-9* Patient Health Questionnaire-9, *OASIS* Overall Anxiety Severity and Impairment Scale, *KIMS* Kentucky Inventory of Mindfulness Skills, *OQ-‘45* Outcome Questionnaire 45-item version, *CSQ* The Client Satisfaction Questionnaire, *GAD-7* General Anxiety Disorder-7, *BDI* Beck Depression Inventory, *BSI* Brief Symptom Inventory & GSI, Global Severity Index (mean of all BSI items), *GAF* Global Assessment of Functioning via the *SCID-I* Structured Clinical Interview for DSM-IV Axis I Disorders, *n.s* not statistically significant, *n.c* non calculable, *SITBI* Self-Injurious Thoughts and Behaviors Interview, *OASIS* Overall Anxiety Severity and Impairment Scale, *PHQ-9* Patient Health Questionnaire-9, *OAKQ* Opposite Action Knowledge Questionnaire, *SSI* Scale for Suicide Ideators, *AUDIT* Alcohol Use Disorders Identification Test, *TLFB* Timeline Follow back

^a^every time the DBT Coach was activated questions about emotional intensity (a) and urges to use substances (b) were assessed

^b^short questionnaire at the end of each day to rate their highest urge to use substances (a), how helpful the DBT Coach app (b) and the Opposite Action skill (c) were that day

^c^a 7-item self-report measure Behavioral Confidence Questionnaire

^d^therapists were asked to rate their patients skill use in general (a) and the use of the opposite action skill before and after using the DBT Coach application (b). In addition, they asked therapists to count the number of phone calls outside the sessions 2 weeks before and after the use of de DBT Coach application (c)

#### Efficacy and clinical utility of telecom for between-session-contact, in support of skills generalization (RQ1)

##### Telephone

Looking first at the treatment efficacy, we did not find any RCTs or pre-post studies about the added value of phone consultation in DBT. However, there are articles that contain data on phone consultation frequency and associations with treatment outcome. Chalker et al. [52] investigated the association between phone calls, satisfaction (patients and therapists), and treatment outcome (patients) in a standard DBT program with adults diagnosed with BPD. More frequent between-session contact was significantly associated with client satisfaction, therapist satisfaction, treatment retention and decrease in psychosocial problems. There was no association between violation of personal limits regarding phone calls and any of the outcome measures. Oliveira and Rizvi [53] studied the frequency of phone calls and text messaging in a sample of patients with BPD who were participating in 6 months of standard DBT. Results showed that therapists received an average of 2.55 phone calls a month. Limbrunner et al. [54] investigated DBT phone consultation to reduce eating disorder-related urges in DBT for eating disordered adults. Results indicated that the average number of calls ranged between 0 to 4 calls per day. The duration of the phone consultation range between 1 to 30 min, with an average of 6 min.

The other studies focus on the clinical utility of phone consultation. Linehan formulated most of her expert opinions regarding phone consultation in the DBT treatment manual [10]. In later articles [55, 56] she highlighted aspects of this mode of treatment and substantiates the assumption that DBT reduces the contingency of between-session contact and suicidal behavior. Linehan emphasizes the importance of *timing*, i.e. calling before self-destructive behavior takes place, and *content*, i.e. focused on skills in a ‘matter of fact’ tone. She also asserts that the individual therapist is best placed to provide phone consultation, since it is the only person who can assuage relationship cracks between sessions, knows the patients learning history and current skills, and can discuss the chain of events that led up to the consultation. At the same time, Linehan accentuates the necessity of teaching the patient skillful ways of asking for help (i.e. “making the therapist want to talk to them on the phone”), because it can be life saving for chronic suicidal patients. This requires firm but flexible use of personal limits that may vary by patient, time and context.

In response to the first RCT of Linehan et al. [57], on the effectiveness of DBT, R.E. Hoffman wrote ‘a letter to the editor’ [58] where several comments and questions about the trial were posed, including the comment that for the treatment as usual (TAU) condition, telephone consultation 24/7 is not feasible. Linehan and Heard acknowledged that the availability of therapists probably had been greater in DBT compared to TAU. However, the actual amount of telephone calls per month did not differ. Linehan and Heard also pointed out that there was no correlation between the number of phone calls and the number of parasuicide episodes in DBT, in contrast to TAU, where such a correlation was found.

Implementation studies and expert opinions provide some additional insight in the clinical utility of telephone consultation in DBT. Chugani and Landes [59] conducted a survey amongst clinicians to investigate the implementation of DBT in College Counseling Centers. A frequently reported barrier to implement the standard DBT program was the unwillingness of individual therapists to offer phone consultation. Flynn et al. [60] reported similar findings in a survey exploring challenges experienced by clinical sites of implementation of DBT in community settings. Common issues concerned therapists’ reluctance, lack of management support and issues regarding clinical responsibility. Landes et al. [61, 62] made a step-by-step inventory of the implementation process of DBT in the Veterans Health Administration (VHA) healthcare system. Barriers in the implementation of DBT in a routine setting, rated as ‘unable to overcome’, were all related to phone consultation. In Landes et al. [63], the authors identify four specific challenges and solutions with regard to phone coaching: 1) ‘tools’ such as work telephone, laptop that gives access to the electronical medical records of patients, organizational policies and procedures, 2) compensation for after-hours phone coaching, 3) willingness of clinicians to provide phone coaching and 4) consistent program and leadership support.

Manning [64], Koons [65] and Ben-Porath [66–69], describe how to carry out phone consultations. They emphasize the importance of explaining in detail the essence and the works of phone consultation to the patient, resulting in an agreement. When carefully introduced at forehand, patients will be more aware of the contingencies of problem behavior. Based on clinical experience, myths are confronted that exist about the expected disastrous impact of phone consultation on the therapists’ life and professional career (among others: being called each night, burnout, inadvertently reinforcing maladaptive behavior and thus enhancing suicide risk, risk of being sued). At the same time, common errors (among others: failure to orient patients, errors concerning contingency management, using phone consultation for other needs than skill generalization, contact recovery or encouragement, setting limits when they are not crossed) and the need of support, validation and problem solving in the therapist consultation team are discussed. Finally, Steinberg et al. [70] emphasizes the importance of making informed decisions concerning parental involvement in phone consultation, therefore they wrote a decision-tree to assist in determining when parental involvement is necessary with adolescents.

##### Videoconferencing

Chu et al. [71] describe two case studies on the DBT school refusal (DBT-SR) program. In this program, DBT strategies were used to target emotional and behavioural dysregulation (including internalizing problems like anxiety and depression) in youth that refuse to go to school. DBT for adolescents (DBT-A) [72] served as the foundation for DBT-SR. Chu et al. added Web-Based Coaching (WBC), via video-conferencing, to the traditional option of phone consultation. This way, both youth and parents could be present and the therapist could directly observe and coach interactions among multiple family members. WBC sessions lasted 5 to 30 min and had a flexible format. The frequency depended on the number of school days the youth had missed the previous week. Results of two case studies show that WBC seemed to provide unique value that improved generalizability of skill acquisition and a sense of support. Both youth, parents and therapists commented that WBC helped increase morning structure, provided real-time assessment and encouragement/support, and helped youth and parents practice skills at critical times.

#### Efficacy and clinical utility of telepsychology in other modes of treatment, i.e. individual therapy, skills training, consultation team (RQ2)

##### Internet

We found two controlled trials about the efficacy of internet-delivered DBT skills training. Lungu [44] developed and evaluated the effectiveness of a computerized, transdiagnostic DBT skills training for Emotion Regulation (iDBT-ER) for adults with a wide range of psychopathology. This online intervention consisted of 81-h weekly sessions: the first two sessions focused on mindfulness skills, followed by six sessions on emotion regulation skills. Every session followed the same structure: an overview of session material, mindfulness practice, homework review, teaching new skills (using videos), practicing new skills (variety of online assignments), assigning new homework and anticipating potential obstacles. Results of this pilot were compared to a matched historical control group who received face-to-face DBT skills training (DBT-ST). Participants of the iDBT-ER reported progress in all of the primary outcomes, namely emotion dysregulation, psychopathology (anxiety, depression), general distress, DBT skills practice and mindfulness skills practice. Compared to the historical control group, pre-post effect sizes were similar for skills practice. Pre-post effect sizes were lower for iDBT-ER concerning anxiety, depression and general distress. Compared to DBT-ST, iDBT-ER reported a strong and significant increase in self-reported mindfulness (See Table 1).

Wilks et al. [50, 51, 73, 74] performed an RCT to evaluate the feasibility, acceptability and efficacy of an eight-session internet delivered DBT skills training intervention (iDBT-ST), for suicidal adults who engage in heavy episodic drinking. Each session lasted approximately 30–50 min and included 2–3 new DBT skills. Each skill was introduced via a short video. Finally, participants engaged in interactive and guided practice. At the end of each session, participants selected a homework exercise. Participants received DBT worksheets and were encouraged via daily emails and/or text messaging. One third of all participants completed the training. No clinical differences were found between drop-outs and completers of iDBT-ST (See Table 1). Results showed that technical problems appeared to pose barriers to treatment feasibility and completion. However, over the four-month study period, an immediate and significant reduction in suicidal ideation, alcohol use, alcohol quantity and frequency and emotion regulation compared to the waiting list controls was found, with large effect sizes for suicidal ideation and alcohol consumption.

##### Videoconferencing

Salamin et al. [42] compared the diary card data of seven patients suffering from borderline personality disorder during two periods: 8 weeks prior to confinement (i.e., set of measures to slow down the spread of COVID-19) and during 8 weeks of confinement. From the 16th of March 2020 to the 26th of April 2020, individual therapy, DBT skills training and consultation team meetings were only possible by means of phone consultation or videoconferencing. Moreover, DBT skills training was limited to 45 min (instead of 2 h) per week and was provided individually. Using a multilevel approach, they found a significant decrease in self-reported binge-eating, fear, shame/ guilt and tension even when they switched to videoconferencing. At the same time, problem behaviours including suicidal behaviour, NSSI, anger outbursts, and experiences including sadness, anger, happiness, emptiness and suicidal ideation did not change significantly in this period of time. Self-reported distress increased significantly (See Table 1).

Lopez et al. [43] performed a pilot study comparing group cohesion between patients who participated in a DBT skills training group via Video Teleconferencing (VTC) and an in-person DBT group. The primary diagnosis of the patients was depression but patients with bipolar and anxiety disorders were also included. Results show that the relationship with the facilitator and the feeling of their learning capacity did not differ between the two groups. There was a significant difference on the relation to member interaction and group cohesion between the two groups (Table 1). The VTC group found it harder to connect with each other in the virtual environment. Compared to the in-person DBT group, the VTC group had a significant better attendance although they reported that attending the group via telehealth would not have been their first choice. Treatment via VTC was preferable to no treatment at all.

Concerning the clinical utility, one survey study and one expert opinion was found. Lakeman and Crighton [75] conducted a survey amongst clinicians to explore the impact of the COVID-19 measures on various DBT programmes for patients with BPD and obstacles to engaging with patients and colleagues via online platforms. Results show that the primary obstacles to providing DBT via online platforms were service and clinician centred obstacles. Few clinicians expressed confidence in being able to adapt to online DBT. Clinicians had no experience of using online platforms and some did not have access to internet or privacy in their home environment. The authors concluded that clinicians need to be supported through education, supervision and coaching in the use of telehealth interventions.

O’Hayer [76] highlighted challenges and opportunities of comprehensive DBT for BPD via the online video conferencing platform ZOOM. Challenges in the skills training were that patients tended to get distracted, feel ashamed or disconnect. Opportunities included using the chat function to communicate with participants, spontaneous ‘virtual tours’ during the break and the ability to choose a screen name. Challenges for the individual treatment were less engagement and increased concerns about the therapist being distracted. Overall, patients reported that they felt less connected.

##### Video

Waltz et al. [77] performed an RCT to evaluate the feasibility of a psychoeducational video to learn adults with BPD naïve to DBT a novel DBT skill. In the experimental condition participants viewed the experimental video first and watched the control video after a week (designed to control time, attention and repeated testing). In the control group the order was reversed. Follow up was 1 week later. In the experimental video Marsha Linehan taught ‘opposite action’, part of the emotion regulation module of DBT [78]. Twenty percent of the participants dropped out. All remaining participants used the opposite action skill one or more times in the week after watching the experimental video. At follow-up, a significant reduction in painful emotions was reported as well as an increase in knowledge, increase in expectations of a positive outcome, and high satisfaction after watching the video of opposite action (Table 1).

#### Efficacy and clinical utility of adding telepsychology to standard DBT modes, −strategies, −procedures (RQ3)

##### Mobile (web) application

Four trials provided data on the efficacy and clinical utility of DBT mobile applications. Rodante et al. [46] investigated the acceptability and preliminary effectiveness of an interactive mobile-health application in 18 adults who participated in DBT at least a month and showed suicidal behaviours or NSSI. The app, named CALMA, provided DBT skills and evidence-based tools to prevent suicide. There were four modalities: “out of crisis”, “I need help”, “problem-solving” and “emergency”. The emergency modality provides users with their emergency contacts and the possibility to share their location with others. It is automatically activated if the app detects that distress does not decrease after three attempts to use skills. Bayesian analysis^2^ showed a high probability of decreased suicidal ideation (*p* = .966), suicidal plan (*p* = .849), suicidal gesture (*p* = .760), thoughts about NSSI (*p* = .909) and NSSI (*p* = .826) in the group where CALMA was added to DBT. The authors reported a high probability of a greater decrease in suicidal ideation and NSSI in DBT + CALMA, compared to DBT only. However, all interval of the comparisons included zero. The app also showed good acceptability by users.

Rizvi et al. [47] explored the feasibility of a DBT Coach smartphone app for the emotion regulation skill ‘opposite action’. Adults diagnosed with BPD and a comorbidity with substance abuse disorders, already in standard DBT treatment, received a smartphone with the app for 10–14 days, which they could use whenever needed. The app consisted of instructions on how to apply the opposite action skill, and mindfulness. Each app usage started and ended with a rating on emotional intensity and urges to use drugs on a 0–10 Likert scale. The app was on average used 15 times over the course of 13 days. Participants reported that the use of the app strengthened their knowledge and self-efficacy in execution of the skill. The intensity of the emotions and the urge to abuse substances decreased significantly after usage and finally a decrease in depression, psychological distress and in overall substance use during the trial was found (See Table 1). An extended version of the DBT Coach was evaluated by Rizvi, Hughes and Thomas [48]. The app was used by adults diagnosed with BPD and had a recent history of NSSI. The app was used as an add on to 6 months of standard DBT. The app included skills from mindfulness, emotion regulation, interpersonal effectiveness and distress tolerance. Results showed a reduction in distress and in urges of self-harm directly after using the app. Frequency of use did not correlate with treatment outcomes, except for the frequency of NSSI episodes, where higher use led to fewer episodes (See Table 1). Over 90% of the participants reported that the app was easy to understand and to use, and that they would use the app if available outside the trial.

Schroeder et al. [79] developed “Pocket Skills”, a mobile web app that uses texts, videos and images of Marsha Linehan to teach DBT-skills (i.e. basics, mindfulness, emotion regulation, distress tolerance and addiction). A conversational agent promotes engagement to the app and use of the skills. Pocket Skills also gives access to a DBT diary card day by day. Participants were adults diagnosed with depression, Generalized Anxiety Disorder (GAD), BPD, Post Traumatic Stress Disorder (PTSD) or bipolar disorder. Schroeder et al. conducted a 4-week field study. Participants were randomized in two groups. The experimental group received semi-personalized text messages each morning like ‘*One of your mindfulness goals is to reduce pain, tension and stress! Keep practicing mindfulness skills!*’. The other group received non-personalized text messages. Depression, anxiety and dysfunctional coping decreased, and the use of DBT skills increased (Table 1). Participants who received daily semi-personalised messages practiced more skills, which resulted in faster improvements. However, we found no information about the randomization process and the number of participants in each group. The app was rated ‘very usable’. Exit-survey data confirmed that Pocket Skills helped patients to stay engaged in DBT and practice their skills.

Four articles contained descriptions of clinical utility by opinions and qualitative data on DBT-applications for smartphones and tablets. Austin et al. [80] evaluated a smartphone application that was developed to support DBT. Participants who were receiving DBT used the app. Overall, there was a positive perception of the app’s efficacy and usability. Helweg-Joergensen et al. [49] developed a smartphone application as an adjunct to DBT to fill in DBT diary cards, called mDiary. Adults who were enrolled in active DBT treatment used the app for at least 4 weeks. The diary card data could be consulted by therapists online. The authors concluded that the mDiary App was an acceptable and relevant innovation for both patients and therapists, although patients experienced a better usability than therapists. Cristol [81] describes the perspective of one BPD patient in DBT on how using technology in DBT can be validating and helpful in discovering typical responses to common stressors. This patient tried several mobile applications and found ‘Daylio’ most useful. The usage of this app helped this patient to minimize stigma as using a smartphone is a common habit, in contrast to filling our diary cards using paper and pencil. Washburn and Parrish [45] described their experience with the DBT Self-Help mobile application, that gives access to key DBT skill sets such as mindfulness, distress tolerance, emotional regulation and interpersonal effectiveness. They recommended the application for patients already enrolled in a DBT program.

##### Virtual reality

Looking first at the treatment efficacy, Navarro-Haro et al. [82] evaluated the virtual reality Dialectical Behavior Therapy (VR DBT) mindfulness skills training in a pre-post study with patients with a GAD. Patients were randomly assigned to a Mindfulness-Based Intervention (MBI) with or without 10 min of VR DBT Mindfulness skills training. The MBI consisted of seven modules, once a week. Following the first six sessions of the MBI, half of the participants also took part in six individual 10-min sessions of VR DBT mindfulness skills training. During the VR DBT mindfulness skills sessions, a participant made use of a headset, float down a 3D computer-generated river while listening to one of three mindfulness skills training audio tracks. The audio tracks were ‘observing sound’, ‘observing visuals’ and ‘wise mind’. Both groups showed a significant decrease in GAD symptoms (see Table 1). The MBI plus VR DBT group retained significantly more participants than the MBI group alone. Additional pre-post improvements showed that the MBI plus VR DBT group improved in the non-judging facet of mindfulness, the interference subscale of the Emotion Regulation Scale and the state of relaxation after all the VR DBT sessions.

There are three studies concerning the clinical utility of virtual reality. First, Navarro-Haro et al. [83] investigated the clinical utility of virtual reality (VR) by using immersive VR to facilitate mindfulness skills training in DBT, as described above. They wrote a case study of a 32-year-old woman diagnosed with BPD and substance use disorder who received standard DBT. Key measurements were administered before and after each VR DBT mindfulness skills training session and results showed that urges to commit suicide, self-harm, quit therapy, use substances and negative emotions measured by the diary card were all reduced after each VR mindfulness session.

Gomez et al. [84] used the VR DBT mindfulness skills training in a case study of a 21-year-old male with severe skin burn covering one third of his body. The primary assessment consisted of measurements of post-traumatic stress disorder, mindfulness acceptance and positive and negative emotions before and after each VR DBT skills training. Results show that the patient accepted the VR DBT and wanted to continue using mindfulness. There was a small reduction in PTSD symptoms after four VR DBT sessions and the reduction in negative emotions was most pronounced after the first VR DBT session but decreased even more the second time and stayed near zero the third and fourth time. Positive emotions were very high after the VR DBT sessions. The same virtual reality enhanced DBT mindfulness skills training was used by Flores et al. [85]. They describe a case study investigating the feasibility of the virtual reality enhanced DBT (VR DBT) mindfulness skills training for two patients with spinal cord injury. The primary assessment consisted of measurements of depression, anxiety and positive and negative emotions before and after each VR DBT skills training. Results showed that patients not only accepted VR as part of their treatment, but also liked using it. Both patients showed a reduction in ratings of depression, nervousness/anxiety and reported being less emotionally upset after the VR DBT skills training. Patient 1 showed also a reduction of negative emotions, where the negative emotions of patient 2 increased directly after the VR DBT mindfulness skills session.

##### Computer program

Görg et al. [86] examined the acceptance and feasibility of the computer program MORPHEUS, as a part of a Dialectical Behavior Therapy Post Traumatic Stress Disorder (DBT-PTSD) residential treatment, that allows computer-assisted in sensu exposure and exercise in self-management during the treatment of PTSD. MORPHEUS can be used to record, and listen to the recordings of, in sensu exposure sessions. Meanwhile playing the recorded sessions patients monitor their level of distress and state dissociation. If the level of state dissociation is too high, MORPHEUS offers one of the 15 diverse skills in order to regulate themselves. Patients received a 12-week multicomponent residential treatment based on the principles of DBT. All participants were diagnosed with PTSD and used MORPHEUS as often as it was required in the standard DBT-PTSD protocol, that is, at least 2 to 5 times a week. Results show that patients found the skills helpful to block dissociation and wanted to use the program again in therapy and would recommend this program to a friend. Meanwhile, patients rather used their DBT skills during exposure instead of using the digital skills in MORPHEUS.

## Discussion

We conducted a scoping review to help DBT-therapists make empirically supported decisions about the use of telepsychology during and after the current pandemic and to anticipate the changing digital needs of patients and clinicians.

Our first focus was the efficacy and clinical utility of telepsychology in standard DBT, i.e. using telecom for between-session-contact, in support of skills generalization. The literature provides us with valuable information about using telephone and videoconferencing to support this mode of treatment. Despite the fact that telephone was the first technology that was used to provide between-session coaching, quantitative information on efficacy and utility of phone coaching by the individual therapist remains limited. We found data about frequency of out of session contact [52–54], percentages for which calls are made in telephone consultation [53, 54], associations between out of session contact and decrease in drop out and greater change of psychological symptoms [52]. However, an RCT about the added value of out of office availability is missing. This is striking, given the function telephone consultation has, the fact that this treatment mode is a key barrier for the implementation of DBT [59–63] and that experts describe how out of office availability places a substantial strain on DBT teams. A RCT in suicidal BPD patients that compares 24/7 access to between-session coaching provided by the individual therapist versus another service/ application versus no between-session coaching is delicate. However, a trial performed by Nadort et al., about the added value of telephone availability in schema focused therapy (SFT) for BPD, suggests that it is feasible [87]. An alternative would be to build on previous work of Oliviera and Rizvi [53] and collect more fine-grained data on the between-sessions contact in ongoing and future DBT –trials. As there is a strong increase of research into DBT [87], a meta-analysis on the subject could be within reach.

Our second focus was to identify research about telepsychology in DBT modes of treatment that are usually provided face-to face (i.e. individual therapy, skills training or consultation team). Quantitative studies on individual therapy or DBT skills training by means of videoconferencing (i.e. synchronous communication with trainers and peers) or blending face-to-face individual therapy with interactive online modules (i.e. asynchronous communication with trainers) are scarce, and still in an early phase of clinical research: evaluating feasibility, acceptance and usability. We did not find RCTs that tested the hypothesis that online or blended DBT is superior or at least equally effective as standard, face-to-face DBT. At the same time, we observe a steady increase of online or blended care in clinical practice, with the coronavirus pandemic as a catalyst [7, 26, 27]. In the absence of sufficient evidence, we think it is advisable to return to face-to-face contact as soon as possible and to remain aware of selection bias, confirmation bias and technology optimism. Cautioning against bias is not the same as stating that online or blended DBT is not efficacious or useless. Despite the concerns and challenges that were reported (i.e. technical issues, difficulties to achieve connectedness and group cohesion [43, 74, 75], we found no reports of serious adverse events or loss of learning capacity in teams that switched to videoconferencing-platforms during the pandemic [42, 74, 75]. Treatment via videoconferencing was preferable to no treatment at all [74]. In addition, the results of pre-post studies about acquiring DBT-skills by means of internet-delivered modules [44, 50, 51, 73] and the psychoeducational video [76] are promising. The next step is to test the efficacy of online or blended DBT in trials with more sophisticated methodologies, and to understand what works for whom, why and in what circumstances. Recent RCTs that were performed in the adjacent field of telepsychology in DBT-trainings and -supervision for clinicians can be a source of inspiration [19–24].

The third and last focus was to review the efficacy or clinical utility of adding telepsychology to standard DBT modes, −strategies, −procedures and skills. We did not find a trial that investigated the added value of standard DBT + telepsychology in comparison to standard DBT. However, smartphone apps are experienced as an acceptable facilitator in access and implantation of DBT skills and led to decrease of a broad range of psychopathology [46–48, 78–80]. One patient described how usage of an application could help to minimalize stigma, as the use of a smartphone is experienced as a common habit [72]. The advantages of a mobile application is that daily personal (text) messages can be added to support using the app. Individuals who received such daily semi-personalised messages practice more skills than those who do not, which results in faster improvement [48]. Future applications could extend current functionalities, for example by creating tailored and gamified content, easily accessible at the right place and time [88]. It is also worth investigating to what extent advanced mobile applications could be a assistive technology for DBT therapists, especially in the implementation of out of office availability. Furthermore, we could add the use of wearables to passively monitor patients’ state and become even more effective in orienting patients to DBT skills when they need them the most [89, 90]. In line with the positive experiences of using mobile applications, the preliminary results of using VR to facilitate mindfulness skills training are positive [45, 82–84]. The usage of VR helped preventing drop-out in GAD patients [45] and two case studies showed that patients liked using VR [83, 84].

In our introduction, we stated that is important to anticipate the needs of future patients and clinicians [90–93]. In performing our scoping review we could not help but wonder whether we aren’t always a couple of steps behind of our youngest patients. For example, we know from clinical practice that patients in DBT skills training groups stay in touch via mobile apps and social media, to share information and to support each other. There are podcasts, youtubers and influencers that discuss DBT-skills. These phenomena may have a larger impact on acquisition and generalization of DBT skills than we think (for better or for worse). At the same time, we know that people build identities and friendships online, and that conflicts, ostracism, bullying and abuse are increasingly taking place in virtual environments. Maybe it is time to add worksheets about ‘digital technology skills’ in the next DBT skills manual? The point here is that, more than ever, we think that it is wise to consult with our patients, younger peers and experts in adjacent fields if we want to remain accessible, effective and relevant in a digital age.

## Conclusion

A shift towards videoconferencing and online trainings is justifiable if it is the only way to get an evidence-based treatment like DBT to patients that need it. However, current research evidence does not support a permanent shift towards online or blended DBT. It is pivotal and timely to increase efforts to investigate the efficacy of online/ blended DBT, compared to standard face-to-face DBT. In addition, we need to gain insight into the benefits of out-of-office availability (e.g. ‘phone consultation) as a standard module of DBT for suicidal patients. Lastly, other technologies should continue to be explored, as smartphone applications, virtual reality, social media platforms, podcasts, semi-automated online communication and more, all hold promise for assessment, skill acquisition and generalization. We need to move forward on this, to improve both the range and effectiveness of existing approaches, to address the high demand for professional support and to anticipate the needs of clinicians and patients with emotion regulation disorders.

## Supplementary Information

**Additional file 1.** Search terms for Pubmed, Embase, PsycArticles and Web of Science.

**Additional file 2.** Syntax for Pubmed, Embase, PsycArticles and Web of Science.

**Additional file 3.** Full-text articles assessed for eligibility and primary reason for exclusion.

## Data Availability

Not applicable.

## Abbreviations

DBT: Dialectical Behavior Therapy
BPD: Borderline Personality Disorder
NSSI: Non Suicidal Self Injury
RCT: Randomized controlled trials
TAU: Treatment As Usual
VHA: Veterans Health Administration
iDBT-ER: Internet Dialectical Behavior Therapy for Emotion Regulation
DBT-ST: Dialectical Behavior Therapy Skills Training
iDBT-ST: Internet Dialectical Behavior Therapy Skills Training
VTC: Video Teleconferencing
DBT-SR: Dialectical Behavior Therapy school refusal
WBC: Web-Based Coaching
VR DBT: Virtual reality Dialectical Behavior Therapy
GAD: Generalized Anxiety Disorder
MBI: Based Intervention
DBT-PTSD: Dialectical Behavior Therapy Post Traumatic Stress Disorder
SFT: Schema Focused Therapy
ESM: Experience Sampling Method

## Footnotes

1 Standard terminology is lacking as yet [1], reason why we use the term telepsychology to broadly refer to concepts such as e-health, telemedicine, telepsychiatry, digital health, teletherapy.

2 Because of the difference between ‘classical’ null-hypothesis significance testing and Bayesian procedures, we decided to cite the primary outcomes of Rodante et al. in the text instead of Table 1
