# Supplementary material for: Reviewing the availability, efficacy and clinical utility of Telepsychology in dialectical behavior therapy (Tele-DBT)
Source: Borderline Personal Disord Emot Dysregul. 2021 Oct 30;8:26. doi: 10.1186/s40479-021-00165-7 (PMC8556811; doi:10.1186/s40479-021-00165-7)
Supplement: Supplementary file 1 — Additional file 1. Search terms for Pubmed, Embase, PsycArticles and Web of Science. [file 40479_2021_165_MOESM1_ESM.docx]

**Additional file 1:**

**Search terms for Pubmed, Embase, PsycArticles and Web of Science**

| **Concept** | **MeSH (Pubmed)** | **Emtree (Embase)** | **Thesaurus (PsycArticles)** | **Text** |
| --- | --- | --- | --- | --- |
|  |  |  |  |  |
| Telepsychology | Telemedicine Telephone  Electronic mail  Videoconferencing  Blogging  Virtual Reality  Augmented Reality  Social Media | Telehealth  Mass communication  Computer simulation | Computer Assisted Therapy  Telephone systems  Information and communication technology  Electronic Health Services | Telecommunication  Telecom  Technology  Technology-based  Computer  Computerized  Internet  Internet-delivered  Internet-based  Online  Web-based  Cyber  Multimedia  Audiovisual  Mobile  mHealth  m-Health  Electronic  Electronically  eHealth  e-health  Telehealth  Tele-health  Teletreatment  Tele-treatment  Teletherapy  Tele-therapy  Telemedicine  Tele-medicine  Video  Videoconferencing  Video-conferencing  Teleconsultation  Tele-consultation  Remote consultation  Intersession contact  Inter-session contact  Between-session contact  Telephone  Phone  Smartphone  Application  App  Telepsychiatry  Tele-psychiatry  Computer-Assisted  Teleconferencing  Tele-conferencing  Telepsychology  Tele-psychology  Telerehabilitation  Tele-rehabilitation  Email  E-mail  Webcast  Podcast  Really Simple Syndication  RSSStreaming  Blogging  Blog  Virtual  Augmented  Social media |
| Dialectical Behavior Therapy | Dialectical Behavior Therapy | dialectical behavior therapy | Dialectical behavior therapy | Dialectical Behavior Therapy  Behavior Therapy, Dialectical  dialectical behavior treatment dialectical behavioral therapy dialectical behavioral treatment  dialectical behaviour therapy  dialectical behavioural therapy |
