# Supplementary material for: Reviewing the availability, efficacy and clinical utility of Telepsychology in dialectical behavior therapy (Tele-DBT)
Source: Borderline Personal Disord Emot Dysregul. 2021 Oct 30;8:26. doi: 10.1186/s40479-021-00165-7 (PMC8556811; doi:10.1186/s40479-021-00165-7)
Supplement: Supplementary file 2 — Additional file 2. Syntax for Pubmed, Embase, PsycArticles and Web of Science. [file 40479_2021_165_MOESM2_ESM.docx]

**Additional file 2:**

**Syntax for Pubmed, Embase, PsycArticles and Web of Science**

**Pubmed Search 13**(((Dialectical Behavior Therapy[Title/Abstract] OR Behavior Therapy, Dialectical[Title/Abstract] OR Dialectical Behaviour Therapy[Title/Abstract] OR Behaviour Therapy, Dialectical[Title/Abstract]) OR dialectical behavior treatment[Title/Abstract] OR behavior treatment, dialectical[Title/Abstract] OR dialectical behaviour treatment[Title/Abstract] OR behaviour treatment, dialectical[Title/Abstract] OR dialectical behavioral therapy[Title/Abstract] OR behavioral therapy, dialectical[Title/Abstract] OR dialectical behavioral treatment[Title/Abstract] OR behavioral treatment, dialectical[Title/Abstract] OR dialectical behavioural therapy[Title/Abstract] OR behavioural therapy, dialectical[Title/Abstract] OR dialectical behavioural treament[Title/Abstract] OR behavioural treatment, dialectical[Title/Abstract]) AND (Telemedicine[MesH] OR Telephone[MesH] OR Electronic mail[MesH] OR Videoconferencing[MesH] OR Blogging[MesH] OR Virtual Reality[MesH] OR Augmented Reality[MesH] OR Social Media[MesH] OR Telecommunication[Title/Abstract] OR Telecom[Title/Abstract] OR Technology[Title/Abstract] OR Technology-based[Title/Abstract] OR Computer[Title/Abstract] OR Computerized[Title/Abstract] OR Internet[Title/Abstract] OR Internet-delivered[Title/Abstract] OR Internet-based[Title/Abstract] OR Online[Title/Abstract] OR Web-based[Title/Abstract] OR Cyber[Title/Abstract] OR Multimedia[Title/Abstract] OR Audiovisual[Title/Abstract] OR Mobile [Title/Abstract] OR Health, Mobile[Title/Abstract] OR mHealth[Title/Abstract] OR m-Health[Title/Abstract] OR Telehealth[Title/Abstract] OR Tele-health[Title/Abstract] OR eHealth[Title/Abstract] OR e-health[Title/Abstract] OR Electronic[Title/Abstract] OR Electronically[Title/Abstract] OR Teletreatment[Title/Abstract] OR Tele-treatment[Title/Abstract] OR Teletherapy[Title/Abstract] OR Tele-therapy[Title/Abstract] OR Telemedicine[Title/Abstract] OR Tele-medicine[Title/Abstract] OR Video[Title/Abstract] OR Videoconferencing[Title/Abstract] OR Video-conferencing[Title/Abstract] OR Teleconsultation[Title/Abstract] OR Tele-consultation[Title/Abstract] OR Remote consultation[Title/Abstract] OR Intersession contact[Title/Abstract] OR Inter-session contact[Title/Abstract] OR Between-session contact[Title/Abstract] OR Telephone[Title/Abstract] OR Phone[Title/Abstract] OR Smartphone[Title/Abstract] OR Application[Title/Abstract] OR App[Title/Abstract] OR Telepsychiatry[Title/Abstract] OR Tele-psychiatry[Title/Abstract] OR Computer-Assisted[Title/Abstract] OR Teleconferencing[Title/Abstract] OR Tele-conferencing[Title/Abstract] OR Telepsychology[Title/Abstract] OR Tele-psychology[Title/Abstract] OR Telerehabilitation[Title/Abstract] OR Tele-rehabilitation[Title/Abstract] OR Email[Title/Abstract] OR E-mail[Title/Abstract] OR Webcast[Title/Abstract] OR Podcast[Title/Abstract] OR Really Simple Syndication[Title/Abstract] OR RSS[Title/Abstract] OR Streaming[Title/Abstract] OR Blogging[Title/Abstract]Blog OR Virtual[Title/Abstract] OR Augmented[Title/Abstract] OR Social media[Title/Abstract])

**Embase (Ovid) 286**

('dialectical behavior therapy'/exp OR 'dialectical behavior therapy' OR ((dialectical NEAR/2 behavior*):ti,ab) OR ((dialectical NEAR/2 behaviour*):ti,ab) OR ((dialectical NEAR/2 therapy):ti,ab) OR ((dialectical NEAR/2 treatment):ti,ab) AND ('telehealth'/de OR 'mass communication'/de OR 'computer simulation'/de OR ‘telecommunication':ti,ab OR ‘telecom':ti,ab OR ‘technology':ti,ab OR ‘technology-based':ti,ab OR ‘computer':ti,ab OR ‘computerized':ti,ab OR ‘internet':ti,ab OR ‘internet-delivered':ti,ab OR ‘internet-based':ti,ab OR ‘online':ti,ab OR ‘web-based':ti,ab OR ‘cyber':ti,ab OR ‘multimedia':ti,ab OR ‘audiovisual':ti,ab OR ‘mobile':ti,ab OR ‘mHealth':ti,ab OR ‘m-Health':ti,ab OR ‘electronic':ti,ab OR ‘electronically':ti,ab OR ‘ehealth':ti,ab OR ‘e-health':ti,ab OR ‘telehealth':ti,ab OR ‘tele-health':ti,ab OR ‘teletreatment':ti,ab OR ‘tele-treatment':ti,ab OR ‘teletherapy':ti,ab OR ‘tele-therapy':ti,ab OR ‘telemedicine':ti,ab OR ‘tele-medicine':ti,ab OR ‘video':ti,ab OR ‘videoconferencing':ti,ab OR ‘video-conferencing':ti,ab OR ‘teleconsultation':ti,ab OR ‘tele-consultation':ti,ab OR ‘remote consultation':ti,ab OR ‘intersession contact':ti,ab OR ‘inter-session contact':ti,ab OR ‘between-session contact':ti,ab OR ‘telephone ':ti,ab OR ‘phone':ti,ab OR ‘smartphone':ti,ab OR ‘application':ti,ab OR ‘app':ti,ab OR ‘telepsychiatry':ti,ab OR ‘tele-psychiatry':ti,ab OR ‘computer-Assisted':ti,ab OR ‘teleconferencing':ti,ab OR ‘tele-conferencing':ti,ab OR ‘telepsychology':ti,ab OR ‘tele-psychology':ti,ab OR ‘telerehabilitation':ti,ab OR ‘tele-rehabilitation':ti,ab OR ‘email':ti,ab OR ‘e-mail':ti,ab OR ‘webcast':ti,ab OR ‘podcast':ti,ab OR ‘really simple syndication':ti,ab OR ‘rss':ti,ab OR ‘streaming':ti,ab OR ‘blogging':ti,ab OR ‘blog':ti,ab OR ‘virtual':ti,ab OR ‘augmented':ti,ab OR ‘social media':ti,ab)

**PSYCARTICLES 18**

(SU,TI,AB(“Dialectical Behavior Therapy”) OR TI,AB(dialectical behavior* OR dialectical behaviour* OR dialectical treatment OR dialectical therapy)) AND (SU,TI,AB(“computer assisted therapy” OR “telephone systems” OR "information and communication technology” OR “electronic health services”) OR SU,TI,AB(“telecommunication” OR “telecom” OR “technology” OR “technology-based” OR “computer” OR “computerized” OR “internet” OR “internet-delivered” OR “internet-based” OR “online” OR “web-based” OR “cyber” OR “multimedia” OR “audiovisual” OR “mobile” OR “mHealth” OR “m-Health” OR “electronic” OR “electronically” OR “ehealth” OR “e-health” OR “telehealth” OR “tele-health” OR “teletreatment” OR “tele-treatment” OR “teletherapy” OR “tele-therapy” OR “telemedicine” OR “tele-medicine” OR “video” OR “videoconferencing” OR “video-conferencing” OR “teleconsultation” OR “tele-consultation” OR “remote consultation” OR “intersession contact” OR “inter-session contact” OR “between-session contact” OR “telephone “ OR “phone” OR “smartphone” OR “application” OR “app” OR “telepsychiatry” OR “tele-psychiatry” OR “computer-Assisted” OR “teleconferencing” OR “tele-conferencing” OR “telepsychology” OR “tele-psychology” OR “telerehabilitation” OR “tele-rehabilitation” OR “email” OR “e-mail” OR “webcast” OR “podcast” OR “really simple syndication” OR “rss” OR “streaming” OR “blogging” OR “blog” OR “virtual” OR “augmented” OR “social media”))

**Web of Science 487**

#1
TS=(telecommunication OR telecom OR technology OR technology-based OR computer OR computerized OR internet OR internet-delivered OR internet-based OR online OR web-based OR cyber OR multimedia OR audiovisual OR mobile OR mHealth OR m-Health OR electronic OR electronically OR ehealth OR e-health OR telehealth OR tele-health OR teletreatment OR tele-treatment OR teletherapy OR tele-therapy OR telemedicine OR tele-medicine OR video OR videoconferencing OR video-conferencing OR teleconsultation OR tele-consultation OR remote consultation OR intersession contact OR inter-session contact OR between-session contact OR telephone OR phone OR smartphone OR application OR app OR telepsychiatry OR tele-psychiatry OR computer-Assisted OR teleconferencing OR tele-conferencing OR telepsychology OR tele-psychology OR telerehabilitation OR tele-rehabilitation OR email OR e-mail OR webcast OR podcast OR really simple syndication OR rss OR streaming OR blogging OR blog OR virtual OR augmented OR social media)

#2
TS=((dialectical AND behavior*) OR (dialectical AND behaviour*) OR (dialectical AND therapy) OR (dialectical AND treatment))

#1 AND #2
