# Supplementary material for: Reviewing the availability, efficacy and clinical utility of Telepsychology in dialectical behavior therapy (Tele-DBT)
Source: Borderline Personal Disord Emot Dysregul. 2021 Oct 30;8:26. doi: 10.1186/s40479-021-00165-7 (PMC8556811; doi:10.1186/s40479-021-00165-7)
Supplement: Supplementary file 3 — Additional file 3. Full-text articles assessed for eligibility and primary reason for exclusion. [file 40479_2021_165_MOESM3_ESM.docx]

**Additional file 3:**

**Full-text articles assessed for eligibility and primary reason for exclusion**

1. *Chalker SA, Carmel A, Atkins DC, Landes SJ, Kerbrat AH, Comtois KA. Examining challenging behaviors of clients with borderline personality disorder. Behaviour research and therapy. 2015 Dec 1;75:11-9.
2. *Oliveira PN, Rizvi SL. Phone coaching in Dialectical Behavior Therapy: frequency and relationship to client variables. Cognitive behaviour therapy. 2018 Sep 3;47(5):383-96.
3. *Limbrunner, Heidi M, Ben-Porath, Denise D, & Wisniewski, Lucene. (2011). DBT Telephone Skills Coaching With Eating Disordered Clients: Who Calls, for What Reasons, and for How Long? Cognitive and Behavioral Practice, 18(2), 186–195.
4. *Linehan MM, Armstrong HE, Suarez A, Allmon D, Heard HL. Cognitive-behavioral treatment of chronically parasuicidal borderline patients. Archives of general psychiatry. 1991 Dec 1;48(12):1060-4.
5. *Linehan MM, Heard HL. Impact of Treatment Accessibility on Clinical Course of Parasuicidal Patients-in Reply to R.E. Hoffman [letter to the editor]. Archives of General Psychiatry. 1993 Feb 1;50(2):157-8.
6. *Linehan MM. Dialectical behavior therapy and telephone coaching. Cognitive and Behavioral Practice. 2011;2(18):207-8.
7. *Chugani, Carla D, & Landes, Sara J. (2016). Dialectical Behavior Therapy in College Counseling Centers: Current Trends and Barriers to Implementation. Journal of College Student Psychotherapy, 30(3), 176–186.
8. *Flynn, Daniel, Joyce, Mary, Gillespie, Conall, Kells, Mary, Swales, Michaela, Spillane, Ailbhe, Hurley, Justina, Hayes, Aoife, Gallagher, Edel, Arensman, Ella, & Weihrauch, Mareike. (2020). Evaluating the national multisite implementation of dialectical behaviour therapy in a community setting: a mixed methods approach. BMC Psychiatry, 20(1), 235–235.
9. *Landes SJ, Matthieu MM, Smith BN, Trent LR, Rodriguez AL, Kemp J, Thompson C. Dialectical behavior therapy training and desired resources for implementation: Results from a national program evaluation in the veterans health administration. Military Medicine. 2016 Aug 1;181(8):747-52.
10. *Landes SJ, Rodriguez AL, Smith BN, Matthieu MM, Trent LR, Kemp J, Thompson C. Barriers, facilitators, and benefits of implementation of dialectical behavior therapy in routine care: results from a national program evaluation survey in the Veterans Health Administration. Translational Behavioral Medicine. 2017 Dec 1;7(4):832-44.
11. *Landes, Sara J, Matthieu, Monica M, Smith, Brandy N, McBain, Sacha A, & Ray, Elizabeth S. (2021). Challenges and Potential Solutions to Implementing Phone Coaching in Dialectical Behavior Therapy. Cognitive and Behavioral Practice, 28(1), 66–76.
12. *Manning SY. Common errors made by therapists providing telephone consultation in dialectical behavior therapy. Cognitive and Behavioral Practice. 2011 May 1;18(2):178-85.
13. *Koons CR. The role of the team in managing telephone consultation in dialectical behavior therapy: Three case examples. Cognitive and Behavioral Practice. 2011 May 1;18(2):168-77.
14. *Ben-Porath DD. Intersession telephone contact with individuals diagnosed with borderline personality disorder: Lessons from dialectical behavior therapy. Cognitive and Behavioral Practice. 2004 Mar 1;11(2):222-30.
15. *Ben-Porath DD, Koons CR. Telephone coaching in dialectical behavior therapy: A decision-tree model for managing inter-session contact with clients. Cognitive and Behavioral Practice. 2005 Sep 1;12(4):448-60.
16. *Ben-Porath DD. SPECIAL SERIES: Dialectical Behavior Therapy and Telephone Coaching Introduction. Cognitive And Behavioral Practice, 2011 Nov 1;18(2):165-167.
17. *Ben-Porath DD. Orienting clients to telephone coaching in dialectical behavior therapy. Cognitive and Behavioral Practice. 2015 Nov 1;22(4):407-14.
18. *Steinberg JA, Steinberg SJ, Miller AL. Orienting adolescents and families to DBT telephone consultation: Principles, procedures, and pitfalls. Cognitive and Behavioral Practice. 2011 May 1;18(2):196-206.
19. *Rodante DE, Kaplan MI, Olivera Fedi R, Gagliesi P, Pascali A, José Quintero PS, Compte EJ, Perez AI, Weinstein M, Chiapella LC, Daray FM. CALMA, a mobile health application, as an accessory to therapy for reduction of suicidal and non-suicidal self-injured behaviors: a pilot cluster randomized controlled trial. Archives of suicide research. 2020 Oct 17:1-8.
20. *Rizvi SL, Dimeff LA, Skutch J, Carroll D, Linehan MM. A pilot study of the DBT coach: an interactive mobile phone application for individuals with borderline personality disorder and substance use disorder. Behavior therapy. 2011 Dec 1;42(4):589-600.
21. *Rizvi SL, Hughes CD, Thomas MC. The DBT Coach mobile application as an adjunct to treatment for suicidal and self-injuring individuals with borderline personality disorder: A preliminary evaluation and challenges to client utilization. Psychological services. 2016 Nov;13(4):380-8.
22. *Schroeder J, Wilkes C, Rowan K, Toledo A, Paradiso A, Czerwinski M, Mark G, Linehan MM. Pocket skills: A conversational mobile web app to support dialectical behavioral therapy. In Proceedings of the 2018 CHI Conference on Human Factors in Computing Systems. 2018 Apr 21 (pp. 1-15).
23. *Austin SF, Jansen JE, Petersen CJ, Jensen R, Simonsen E. Mobile App Integration Into Dialectical Behavior Therapy for Persons With Borderline Personality Disorder: Qualitative and Quantitative Study. JMIR Mental Health. 2020;7(6):e14913.
24. *Helweg-Joergensen S, Schmidt T, Lichtenstein MB, Pedersen SS. Using a Mobile Diary App in the Treatment of Borderline Personality Disorder: Mixed Methods Feasibility Study. JMIR formative research. 2019;3(3):e12852.
25. *Cristol S. Patient's perspective on using mobile technology as an aid to psychotherapy. JMIR mental health. 2018;5(4):e10015.
26. *Washburn, Micki, & Parrish, Danielle E. (2013). DBT Self-Help Application for Mobile Devices. Journal of Technology in Human Services, 31(2), 175–183.
27. *Wilks CR, Lungu A, Ang SY, Matsumiya B, Yin Q, Linehan MM. A randomized controlled trial of an Internet delivered dialectical behavior therapy skills training for suicidal and heavy episodic drinkers. Journal of affective disorders. 2018 May 1;232:219-28.
28. *Lungu A. Computerized trans-diagnostic dialectical behavior therapy skills training for emotion dysregulation (Doctoral dissertation). University of Washington; 2015.
29. *Wilks CR, Yin Q, Zuromski KL. User Experience Affects Dropout from Internet-Delivered Dialectical Behavior Therapy. Telemedicine and e-Health. 2020 Jun 1;26(6):794-7.
30. *Wilks C, Yin Q, Ang SY, Matsumiya B, Lungu A, Linehan M. Internet-delivered dialectical behavioral therapy skills training for suicidal and heavy episodic drinkers: protocol and preliminary results of a randomized controlled trial. JMIR research protocols. 2017;6(10):e207.
31. *Salamin, Virginie, Rossier, Vanessa, Joye, Daisy, Nolde, Chrystelle, Pierrehumbert, Timothee, Gothuey, Isabelle, & Guenot, Florence. (2021). Adaptations de la therapie comportementale dialectique ambulatoire en periode de pandemie COVID-19 et consequences du confinement sur des patients souffrant d'un etat-limite/Adaptations of an outpatient Dialectical Behavioral Therapy during the COVID-19 pandemic and consequences of the confinement on patients with borderline personality disorder. Annales Médico Psychologiques, 179(2), 131.
32. *Lopez A, Rothberg B, Reaser E, Schwenk S, Griffin R. Therapeutic groups via video teleconferencing and the impact on group cohesion. Mhealth. 2020;6.
33. *Chu BC, Rizvi SL, Zendegui EA, Bonavitacola L. Dialectical behavior therapy for school refusal: Treatment development and incorporation of web-based coaching. Cognitive and Behavioral Practice. 2015 Aug 1;22(3):317-30.
34. *Lakeman, R, & Crighton, J. (2020). The Impact of Social Distancing on People with Borderline Personality Disorder: The Views of Dialectical Behavioural Therapists. Issues in Mental Health Nursing, 1–7.
35. *O'Hayer, C. Virginia. (2021). Building a Life Worth Living During a Pandemic and Beyond: Adaptations of Comprehensive DBT to COVID-19. Cognitive and Behavioral Practice.
36. *Navarro-Haro, Maria V, Hoffman, Hunter G, Garcia-Palacios, Azucena, Sampaio, Mariana, Alhalabi, Wadee, Hall, Karyn, & Linehan, Marsha. (2016). The use of virtual reality to facilitate mindfulness skills training in dialectical behavioral therapy for borderline personality disorder: A case study. Frontiers in Psychology, 7.
37. *Navarro-Haro, María V, Modrego-Alarcón, Marta, Hoffman, Hunter G, López-Montoyo, Alba, Navarro-Gil, Mayte, Montero-Marin, Jesús, García-Palacios, Azucena, Borao, Luis, & García-Campayo, Javier. (2019). Evaluation of a mindfulness-based intervention with and without virtual reality dialectical behavior therapy® mindfulness skills training for the treatment of generalized anxiety disorder in primary care: A pilot study. Frontiers in Psychology, 10, 55–55.
38. *Gomez, Jocelyn, Hoffman, Hunter G, Bistricky, Steven L, Gonzalez, Miriam, Rosenberg, Laura, Sampaio, Mariana, Garcia-Palacios, Azucena, Navarro-Haro, Maria V, Alhalabi, Wadee, Rosenberg, Marta, Meyer, Walter J, & Linehan, Marsha M. (2017). The use of virtual reality facilitates dialectical behavior therapy® "observing sounds and visuals" mindfulness skills training exercises for a Latino patient with severe burns: A case study. Frontiers in Psychology, 8, 1611.
39. *Flores, Araceli, Linehan, Marsha M, Todd, S. Rob, & Hoffman, Hunter G. (2018). The use of virtual reality to facilitate Mindfulness skills training in Dialectical behavioral therapy for spinal cord injury: A case study.
40. *Görg, Nora, Priebe, Kathlen, Deuschel, Tilman, Schüller, Martin, Schriner, Friederike, Kleindienst, Nikolaus, Ludäscher, Petra, Schmahl, Christian, & Bohus, Martin. (2016). Computer-Assisted In Sensu Exposure for Posttraumatic Stress Disorder: Development and Evaluation. JMIR Mental Health, 3(2), e27–e27.
41. *Waltz J, Dimeff LA, Koerner K, Linehan MM, Taylor L, Miller C. Feasibility of using video to teach a dialectical behavior therapy skill to clients with borderline personality disorder. Cognitive and Behavioral Practice. 2009 May 1;16(2):214-22.
42. Dimeff LA, Koerner K, Woodcock EA, Beadnell B, Brown MZ, Skutch JM, Paves AP, Bazinet A, Harned MS. Which training method works best? A randomized controlled trial comparing three methods of training clinicians in dialectical behavior therapy skills. Behaviour Research and Therapy. 2009 Nov 1;47(11):921-30.

🡪 **No DBT treatment**

1. Dimeff LA, Harned MS, Woodcock EA, Skutch JM, Koerner K, Linehan MM. Investigating bang for your training buck: a randomized controlled trial comparing three methods of training clinicians in two core

🡪 **No DBT treatment**

1. Borschmann R, Henderson C, Hogg J, Phillips R, Moran P. Crisis interventions for people with borderline personality disorder. Cochrane Database of Systematic Reviews. 2012(6).
   🡪 **No DBT treatment**
2. Carrotte E, Hartup M, Blanchard M. “It's very hard for me to say anything positive”: A qualitative investigation into borderline personality disorder treatment experiences in the Australian context. Australian Psychologist. 2019 Dec;54(6):526-35.

🡪 **No DBT treatment**

1. Castell E. Negative attitudes towards borderline personality disorder patients in Spanish mental health and primary care services: mHealth, a possible solution?. Aloma: revista de psicologia, ciències de l'educació i de l'esport Blanquerna. 2017;35(2):23-37.
   🡪 **No DBT treatment**
2. Cook PF, Emiliozzi S, Waters C, El DH. Effects of telephone counseling on antipsychotic adherence and emergency department utilization. The American journal of managed care. 2008 Dec;14(12):841-6.
   🡪 **No DBT treatment**
3. Fassbinder E, Hauer A, Schaich A, Schweiger U, Jacob GA, Arntz A. Integration of e‐health tools into face‐to‐face psychotherapy for borderline personality disorder: a chance to close the gap between demand and supply?. Journal of clinical psychology. 2015 Aug;71(8):764-77.

🡪 **No DBT treatment**

1. Frías Á, Solves L, Navarro S, Palma C, Farriols N, Aliaga F, Hernández M, Antón M, Riera A. Technology-Based Psychosocial Interventions for People with Borderline Personality Disorder: A Scoping Review of the Literature. Psychopathology. 2020 Nov 9:1-10.

🡪 **No original data on telecom in DBT**

1. Han J, McGillivray L, Wong QJ, Werner-Seidler A, Wong I, Calear A, Christensen H, Torok M. A mobile health intervention (LifeBuoy app) to help young people manage suicidal thoughts: protocol for a mixed-methods randomized controlled trial. JMIR research protocols. 2020;9(10):e23655.
   🡪 **Not peer reviewed**
2. Helweg-Jørgensen S, Lichtenstein MB, Fruzzetti AE, Dahl CM, Pedersen SS. Daily Self-Monitoring of Symptoms and Skills Learning in Patients With Borderline Personality Disorder Through a Mobile Phone App: Protocol for a Pragmatic Randomized Controlled Trial. JMIR Research Protocols. 2020;9(5):e17737.

🡪 **No original data on telecom in DBT**

1. Ilagan GS, Iliakis EA, Wilks CR, Vahia IV, Choi-Kain LW. Smartphone applications targeting borderline personality disorder symptoms: a systematic review and meta-analysis. Borderline personality disorder and emotion dysregulation. 2020 Dec;7(1):1-5.

🡪 **No original data on telecom in DBT**

1. Linehan MM, Korslund KE, Harned MS, Gallop RJ, Lungu A, Neacsiu AD, McDavid J, Comtois KA, Murray-Gregory AM. Dialectical behavior therapy for high suicide risk in individuals with borderline personality disorder: a randomized clinical trial and component analysis. JAMA psychiatry. 2015 May 1;72(5):475-82.

🡪 **No original data on telecom in DBT**

1. O’Hearn A, Pollard Jr RQ. Modifying dialectical behavior therapy for deaf individuals. Cognitive and Behavioral Practice. 2008 Nov 1;15(4):400-14.

🡪 **No original data on telecom in DBT**

1. Abdelkarim, A, Nagui Rizk, D, Esmaiel, M, & Helal, H. (2016). Social media group parallel to dialectical behavior therapy skills training group, the pros and cons. European Psychiatry, 33(S1), S556–S556.
   🡪 **Not peer reviewed**
2. Alavi, N, Rivera, M, & Gagnon, K. (2015). Dialectical Behavioural Therapy (DBT)? There is an App for That. European Psychiatry, 30, 845–845.
   🡪 **Not peer reviewed**
3. Armey, Michael F. (2012). Ecological Momentary Assessment and Intervention in Nonsuicidal Self-Injury: A Novel Approach to Treatment. Journal of Cognitive Psychotherapy, 26(4), 299–317.
   🡪 **No DBT treatment**
4. Arnold, Trisha, G. Rogers, Brooke, L. Norris, Alyssa, Schierberl Scherr, Anna, Haubrick, Kayla, E. Renna, Megan, Sun, Shufang, M. Danforth, Margaret, T. Chu, Christina, S. Silva, Elizabeth, Whiteley, Laura B, & Pinkston, Megan. (2020). A brief transdiagnostic pandemic mental health maintenance intervention. Counselling Psychology Quarterly, 1–21.
   🡪 **No DBT treatment**
5. Asarnow, Joan Rosenbaum. (2018). Suicide Attempt Prevention: A Technology-Enhanced Intervention for Treating Suicidal Adolescents After Hospitalization. The American Journal of Psychiatry, 175(9), 817–819.
   🡪 **No DBT treatment**
6. Berman, Anne H, Berman, Anne H, Farzanfar, Ramesh, Farzanfar, Ramesh, Kristiansson, Marianne, Kristiansson, Marianne, Carlbring, Per, Carlbring, Per, Friedman, Robert H, & Friedman, Robert H. (2012). Design and Development of a Telephone-Linked Care (TLC) System to Reduce Impulsivity among Violent Forensic Outpatients and Probationers. Journal of Medical Systems, 36(3), 1031–1042.
   🡪 **No DBT treatment**
7. Bishop, Todd M, Maisto, Stephen A, Britton, Peter C, & Pigeon, Wilfred R. (2016). Considerations in the Use of Interactive Voice Recording for the Temporal Assessment of Suicidal Ideation and Alcohol Use. Crisis : the Journal of Crisis Intervention and Suicide Prevention, 37(5), 370–376.

🡪 **No DBT treatment**

1. Carmel, Adam, Villatte, Jennifer L, Zachary Rosenthal, M, Chalker, Samantha, & Comtois, Katherine Anne. (2016). Applying Technological Approaches to Clinical Supervision in Dialectical Behavior Therapy: A Randomized Feasibility Trial of the Bug-in-the-Eye (BITE) Model. Cognitive and Behavioral Practice, 23(2), 221–229.

🡪 **No DBT treatment**

1. Chugani, Carla D, Fuhrman, Barbara, Abebe, Kaleab Z, Talis, Janine, Miller, Elizabeth, & Coulter, Robert W.S. (2020). Wellness and resilience for college and beyond: protocol for a quasi-experimental pilot study investigating a dialectical behaviour therapy skill-infused college course. BMJ Open, 10(6), e036833–e036833.
   🡪 **No original data on telecom in DBT**
2. Dimeff, Linda A, Woodcock, Eric A, Harned, Melanie S, & Beadnell, Blair. (2011). Can Dialectical Behavior Therapy Be Learned in Highly Structured Learning Environments? Results From a Randomized Controlled Dissemination Trial. Behavior Therapy, 42(2), 263–275.
   🡪 **No DBT treatment**
3. Dimeff, Linda A, Jobes, David A, Chalker, Samantha A, Piehl, Brian M, Duvivier, Leticia Lobo, Lok, Benjamin C, Zalake, Mohan S, Chung, Julie, & Koerner, Kelly. (2020). A novel engagement of suicidality in the emergency department: Virtual Collaborative Assessment and Management of Suicidality. General Hospital Psychiatry, 63, 119–126.
   🡪 **No DBT treatment**
4. Jacob, K. L. (n.d.). Clinical Observations About the Potential Benefits and Pitfalls of Between-Session Contacts with Borderline Patients. Harv Rev Psychiatry, 24(5), e8–e14.
   🡪 **Not peer reviewed**
5. Study analyzing the use of the dialectical behavior therapy skills set and psychosocial stress with breast cancer patients
   🡪 **No original data on telecom in DBT**
6. Kaess, Michael, Koenig, Julian, Bauer, Stephanie, Moessner, Markus, Fischer-Waldschmidt, Gloria, Mattern, Margarete, Herpertz, Sabine C, Resch, Franz, Brown, Rebecca, In-Albon, Tina, Koelch, Michael, Plener, Paul L, Schmahl, Christian, & Edinger, Alexandra. (2019). Self-injury: Treatment, Assessment, Recovery (STAR): Online intervention for adolescent non-suicidal self-injury - Study protocol for a randomized controlled trial. Trials, 20(1), 425–425.
   🡪 **Not peer reviewed**
7. Kauth, Michael R, Adler, Geri, McCandless, Stephen J, & Leopolous, Wendy S. (2017). Embedding new clinical practices: the role of facilitation in enhancing web-based training for mental health providers. The Journal of Mental Health Training, Education, and Practice, 12(1), 24–32.
   🡪 **No DBT treatment**
8. Kenny, Therese E, Carter, Jacqueline C, & Safer, Debra L. (2020). Dialectical behavior therapy guided self-help for binge-eating disorder. Eating Disorders, 28(2), 202–211.
   🡪 **No original data on telecom in DBT**
9. Kerr, Patrick L, Muehlenkamp, Jennifer J, & Larsen, Margo Adams. (2009). Implementation of DBT-Informed Therapy at a Rural University Training Clinic: A Case Study. Cognitive and Behavioral Practice, 16(1), 92–100.
   🡪 **No DBT treatment**
10. Kolar, David Raphael, Bürger, Arne, Hammerle, Florian, & Jenetzky, Ekkehart. (2014). Aversive tension of adolescents with anorexia nervosa in daily course: a case-controlled and smartphone-based ambulatory monitoring trial. BMJ Open, 4(4), e004703–e004703.
    🡪 **No DBT treatment**
11. Kolar, David R, Hammerle, Florian, Jenetzky, Ekkehart, & Huss, Michael. (2017). Smartphone-Enhanced Low-Threshold Intervention for adolescents with Anorexia Nervosa (SELTIAN) waiting for outpatient psychotherapy: study protocol of a randomised controlled trial. BMJ Open, 7(10), e018049–e018049.
    🡪 **No DBT treatment**
12. Lungu, Anita, Jun, Janie Jihee, Azarmanesh, Okhtay, Leykin, Yan, & Chen, Connie E-Jean. (2020). Blended Care-Cognitive Behavioral Therapy for Depression and Anxiety in Real-World Settings: Pragmatic Retrospective Study. Journal of Medical Internet Research, 22(7), e18723–e18723.
    🡪 **No DBT treatment**
13. Masson, Philip C, von Ranson, Kristin M, Wallace, Laurel M, & Safer, Debra L. (2013). A randomized wait-list controlled pilot study of dialectical behaviour therapy guided self-help for binge eating disorder. Behaviour Research and Therapy, 51(11), 723–728.

🡪 **No original data on telecom in DBT**

1. Lessons Learned: Virtual DBT from Therapist and Client Perspectives Poster | Affiche
   🡪 **Not peer-reviewed**
2. Stallard, Paul, Porter, Joanna, & Grist, Rebecca. (2016). Safety, Acceptability, and Use of a Smartphone App, BlueIce, for Young People Who Self-Harm: Protocol for an Open Phase I Trial. JMIR Research Protocols, 5(4), e217–e217.

🡪 **No DBT treatment**

1. Wisniewski, Lucene, & Ben‐Porath, Denise D. (2005). Telephone skill‐coaching with eating‐disordered clients: clinical guidelines using a DBT framework. European Eating Disorders Review, 13(5), 344–350.

🡪 **No original data on telecom in DBT**

1. Worrall, John M, & Fruzzetti, Alan E. (2009). Ismproving peer supervisor ratings of therapist performance in dialectical behavior therapy: An internet-based training system. Psychotherapy (Chicago, Ill.), 46(4), 476–479.

🡪 **No DBT treatment**

1. Wright, Kim, Palmer, Gemma, Javaid, Mahmood, Mostazir, Mohammod, & Lynch, Tom. (2020). Psychological therapy for mood instability within bipolar spectrum disorder: a single-arm feasibility study of a dialectical behaviour therapy-informed approach. Pilot and Feasibility Studies, 6(1), 46–46.

🡪 **No original data on telecom in DBT**

1. McCay, Elizabeth, Carter, Celina, Aiello, Andria, Quesnel, Susan, Howes, Carol, Beanlands, Heather, Langley, John, MacLaurin, Bruce, Hwang, Steven, Cooper, Linda, & Lord, Christina. (2017). Training frontline community agency staff in dialectical behaviour therapy: building capacity to meet the mental health needs of street-involved youth. The Journal of Mental Health Training, Education, and Practice, 12(2), 121–132.

🡪 **No DBT treatment**

1. Navarro-Haro, María V, López-del-Hoyo, Yolanda, Campos, Daniel, Linehan, Marsha M, Hoffman, Hunter G, García-Palacios, Azucena, Modrego-Alarcón, Marta, Borao, Luis, & García-Campayo, Javier. (2017). Meditation experts try Virtual Reality Mindfulness: A pilot study evaluation of the feasibility and acceptability of Virtual Reality to facilitate mindfulness practice in people attending a Mindfulness conference. PloS One, 12(11), e0187777–e0187777.

🡪 **No DBT treatment**

1. Dol, A, Bode, C, Velthuijsen, H, Strien, T. van, & Gemert-Pijnen, J.E.W.C. van. (2021). Application of three different coaching strategies through a virtual coach for people with emotional eating: A vignette study. The International Journal of Eating Disorders, 9.

🡪 **No DBT treatment**
